# The Double Burden of Obesity and Malnutrition in a Protracted Emergency Setting: A Cross-Sectional Study of Western Sahara Refugees

**DOI:** 10.1371/journal.pmed.1001320

**Published:** 2012-10-02

**Authors:** Carlos S. Grijalva-Eternod, Jonathan C. K. Wells, Mario Cortina-Borja, Nuria Salse-Ubach, Mélody C. Tondeur, Carmen Dolan, Chafik Meziani, Caroline Wilkinson, Paul Spiegel, Andrew J. Seal

**Affiliations:** 1 Centre for International Health and Development, UCL Institute of Child Health, London, United Kingdom; 2 Emergency Nutrition Network, Oxford, United Kingdom; 3 Medical Research Council Childhood Nutrition Research Centre, UCL Institute of Child Health, London, United Kingdom; 4 Medical Research Council Centre of Epidemiology for Child Health, UCL Institute of Child Health, London, United Kingdom; 5 Independent Consultant, Barcelona, Spain; 6 Tindouf Sub-Office, United Nations High Commissioner for Refugees, Tindouf, Algeria; 7 Public Health and HIV Section, Division of Programme Support and Management, United Nations High Commissioner for Refugees, Geneva, Switzerland; Epicentre, France

## Abstract

**Background:**

Households from vulnerable groups experiencing epidemiological transitions are known to be affected concomitantly by under-nutrition and obesity. Yet, it is unknown to what extent this double burden affects refugee populations dependent on food assistance. We assessed the double burden of malnutrition among Western Sahara refugees living in a protracted emergency.

**Methods and Findings:**

We implemented a stratified nutrition survey in October–November 2010 in the four Western Sahara refugee camps in Algeria. We sampled 2,005 households, collecting anthropometric measurements (weight, height, and waist circumference) in 1,608 children (6–59 mo) and 1,781 women (15–49 y). We estimated the prevalence of global acute malnutrition (GAM), stunting, underweight, and overweight in children; and stunting, underweight, overweight, and central obesity in women. To assess the burden of malnutrition within households, households were first classified according to the presence of each type of malnutrition. Households were then classified as undernourished, overweight, or affected by the double burden if they presented members with under-nutrition, overweight, or both, respectively.

The prevalence of GAM in children was 9.1%, 29.1% were stunted, 18.6% were underweight, and 2.4% were overweight; among the women, 14.8% were stunted, 53.7% were overweight or obese, and 71.4% had central obesity. Central obesity (47.2%) and overweight (38.8%) in women affected a higher proportion of households than did GAM (7.0%), stunting (19.5%), or underweight (13.3%) in children. Overall, households classified as overweight (31.5%) were most common, followed by undernourished (25.8%), and then double burden–affected (24.7%).

**Conclusions:**

The double burden of obesity and under-nutrition is highly prevalent in households among Western Sahara refugees. The results highlight the need to focus more attention on non-communicable diseases in this population and balance obesity prevention and management with interventions to tackle under-nutrition.

*Please see later in the article for the Editors' Summary*

## Abstract 2

Surveying women and children from refugee camps in Algeria, Carlos Grijalva-Eternod and colleagues find high rates of obesity among women as well as many undernourished children, and that almost a quarter of households are affected by both undernutrition and obesity.

## Editors' Summary

**Background:**

Good nutrition is essential for human health and survival. Insufficient food intake causes under-nutrition, which increases susceptibility to infections; intake of too much or inappropriate food, in particular in interaction with sedentary behaviour, can lead to obesity, which increases the risk of non-communicable diseases such as diabetes. During the past 30 years, the prevalence (the proportion of a population affected by a condition) of obesity has greatly increased, initially among adults in industrialized countries, but more recently among children and in less-affluent populations. Now, worldwide, overweight people outnumber under-nourished people. Furthermore, some populations are affected by both under-nutrition and obesity, forms of malnutrition that occur when the diet is suboptimal for health. So, for example, a child can be both stunted (short for his or her age, an indicator of long-term under-nutrition) and overweight (too heavy for his or her age). The emergence of this double burden of malnutrition has been attributed to the nutrition transition—the rapid move because of migration or urbanization to a lifestyle characterized by low levels of physical activity and high consumption of refined, energy-dense foods—without complete elimination of under-nutrition.

**Why Was This Study Done?:**

Refugees are one group of people in whom under-nutrition and obesity sometimes coexist. Worldwide, in 2010, 15.4 million refugees were dependent on host governments and international humanitarian agencies for their food security and well-being. It is essential that these governments and organizations provide appropriate food assistance programs to refugees—policies that are appropriate during acute emergencies may not be appropriate in protracted emergencies and may contribute to the emergence of the double burden of malnutrition among refugees. Unfortunately, the extent to which the double burden of malnutrition affects refugees in protracted emergencies is unknown. In this cross-sectional study (an investigation that looks at the characteristics of a population at a single time), the researchers assessed the double burden of malnutrition among people from Western Sahara who have been living in four refugee camps near Tindouf city, Algeria, since 1975.

**What Did the Researchers Do and Find?:**

The researchers used data from a 2010 survey that measured the height and weight of children and the height, weight, and waist circumference of women living in 2,005 households in the Algerian refugee camps. For the children, they estimated the prevalence of global acute malnutrition (which includes thin, “wasted” children, as indicated by a low weight for height based on the World Health Organization growth standards, and those with nutritional oedema), stunting, and underweight and overweight (low and high weight for age and gender, respectively). For the women, they estimated the prevalence of stunting, underweight (body mass index less than 18.5 kg/m^2^), overweight (body mass index greater than 25 kg/m^2^), and central obesity (a waist circumference of more than 80 cm). Among the children, 9.1% had global acute malnutrition, 29.1% were stunted, 8.6% were underweight, and 2.4% were overweight. Among the women, 14.8% were stunted, 53.7% were overweight, and 71.4% had central obesity. Notably, central obesity and overweight in women affected more households than global acute malnutrition, stunting, and underweight in children. Finally, based on whether a household included members with under-nutrition or overweight, alone or in combination, the researchers classified a third of households as overweight, a quarter as undernourished, and a quarter as affected by the double burden of malnutrition.

**What Do These Findings Mean?:**

These findings indicate that there is a high prevalence of the double burden of malnutrition among households in Western Saharan refugee camps in Algeria. Although this study provides no information on men and does not investigate whether the obesity seen in these camps leads to an increased risk of diabetes and other non-communicable diseases, these findings have several important implications for the provision of food assistance and care for protracted humanitarian emergencies. For example, they highlight the need to promote long-term food security and to improve nutrition adequacy and food diversity in protracted emergencies. In addition, they suggest that current food assistance programs that are suitable for acute emergencies may not be suitable for extended emergencies. They also highlight the need to focus more attention on non-communicable diseases in refugee camps and to develop innovative ways to provide obesity prevention and management in these settings. However, as the researchers stress, careful policy and advocacy work is essential to ensure that efforts to deal with the threat of obesity among refugees do not jeopardize support for life-saving food assistance programs for refugees.

**Additional Information:**

Please access these websites via the online version of this summary at http://dx.doi.org/10.1371/journal.pmed.1001320.

- Wikipedia provides background information about the Western Sahara refugee camps near Tindouf, Algeria (note that Wikipedia is a free online encyclopedia that anyone can edit)
- The World Health Organization provides information on all aspects of nutrition and obesity (in several languages)
- The United Nations World Food Programme is the world's largest humanitarian agency fighting hunger worldwide; its website provides detailed information about hunger and information about its work in the Western Sahara refugee camps in Algeria, including personal stories and photographs of food distribution
- The United Nations High Commissioner for Refugees is the United Nations body mandated to lead and coordinate international action to protect refugees and resolve refugee problems worldwide; its website provides detailed information about its work in the Western Sahara refugee camps in Algeria
- Oxfam also provides detailed information about its work in the Algerian refugee camps, a description of the camps, and personal stories from people living in the camps
- An article published by the Food and Agriculture Organization of the United Nations explains the double burden of malnutrition

## Introduction

In 2010, the United Nations High Commissioner for Refugees (UNHCR) reported a worldwide estimate of 43.7 million displaced persons [1]; of these, 15.4 million were refugees, as they had crossed an international border. The food security and well-being of refugees falls under the remit of host governments and international organisations like UNHCR and the United Nations World Food Programme. The dynamic and complex setting in which refugees and internally displaced persons live has been reviewed recently and recommendations made [2]. The current context is characterised by an increase in internally displaced persons and a decrease in the number of refugees, a growing proportion of refugees living in urban rather than camp settings, and a worldwide trend towards fewer but longer-lasting conflicts.

During the last 30 years obesity has increased worldwide [3] to the extent that those overweight now outnumber those with under-nutrition [4],[5]. By 2008, 1.46 billion adults were estimated to be overweight; of which 502 million were obese [6]. A World Health Organization expert commission in 2004 calculated the global burden of disease attributable to overweight in adults to be more than 30 million disability-adjusted life years [7]. Although the obesity epidemic was first described in adults from industrialised countries, it is now affecting children, and increasingly affects less-affluent populations [6],[8]. Furthermore, there is growing recognition of a “double burden” of malnutrition among populations in both affluent and less-affluent countries [9], i.e., the coexistence of under-nutrition (e.g., stunting or underweight) with overweight, which has been observed at national and household levels [10],[11]. In its most severe form, the double burden manifests within individuals, for example, as stunted overweight children [12]. The emergence of this double burden is attributed to the nutrition transition that commonly follows rapid economic development, characterised by rapid secular trends (e.g., migration and urbanisation) leading to low levels of physical activity and a high consumption of refined, energy-dense foods, in the absence of the full elimination of under-nutrition [9].

The presence of obesity among refugees, generally assumed to be rare, has nevertheless been described in populations fleeing conflict zones where economic development has already impacted dietary intakes and activity patterns. For example, female Bosnian refugees granted asylum in Sweden had significantly higher values of body mass index (BMI) and waist circumference (WC) than age-matched Swedish women [13]. Likewise, 24.6% of Iraqi refugees, aged >24 y, who resettled in California presented with obesity during their standard refugee medical assessment [14]. The coexistence of under-nutrition and obesity at the population level has likewise been described for urban and rural displaced people living in Armenia and for school children in the Occupied Palestinian Territory, where diabetes mellitus and hypertension in adults were also observed [15],[16].

Refugee populations from less-developed countries that migrate to camps are known to present with nutritional deficiencies, such as iron deficiency anaemia [17]. However, those in stable protracted settings may also experience an epidemiological transition whereby diet and lifestyle change significantly, despite the lack of exposure to economic development, and now such refugees have a longer life expectancy and increased incidence of non-communicable diseases (NCDs) [2]. At present, little is known about whether under-nutrition and overweight coexist among refugees in protracted settings, or about the proportion of households that may be affected by this double burden. This study aimed to use anthropometric data from a routine UNHCR nutrition survey to investigate the existence of the double burden of malnutrition in a refugee population highly dependent upon food assistance and living in a protracted emergency.

## Methods

### Ethics Statement

UNHCR routinely monitors health and nutrition indicators of children aged <5 y and women of childbearing age (15–49 y) in the Western Sahara refugee setting through nutrition surveys. Results are primarily used to better evaluate needs and allocate resources; they are also used to indirectly estimate the impact of programme implementation by assessing secular changes in health and nutrition indicators.

The nutrition survey used in this study, besides estimating nutrition indicators, also aimed to provide pre-intervention baseline data for a future impact evaluation of a UNHCR-led micronutrient supplementation programme intended to reduce micronutrient deficiencies.

When considering whether ethical approval would be required for the impact evaluation, we were guided by a recent expert meeting report [18] that considered that no ethical clearance is required for cross-sectional data collection that is part of routine programme monitoring and that does not collect personal identifier information.

The nutrition survey was approved by UNHCR and the refugee health authorities. Informed verbal consent was obtained from all adults and caregivers.

### Study Population

Since 1975, people from Western Sahara (also known as Sahrawi) have lived as refugees in camps near Tindouf city, in southwest Algeria, an area with a harsh desert environment. Their situation is considered a protracted emergency, as there is a stalemate to negotiations, with no sign of imminent resolution. Although accurate estimates are not available, the host country estimates that there are ∼165,000 people living in four camps (Awserd, Dakhla, Laayoune, and Smara), mostly dependent on food assistance from international organisations.

### Survey Design

A two-stage household cluster survey with four strata (one per camp) was conducted in October–November 2010 to collect nutrition indicators from children (<5 y) and women of childbearing age (15–49 y). The survey design followed UNHCR survey guidelines [19].

### Calculation of Sample Size, Number of Households Required, and Cluster Size

Sample size was calculated using previous prevalence data for global acute malnutrition (GAM), stunting, and anaemia in children, and anaemia and obesity prevalence in women, using ENA for SMART software (beta version, November 2008; http://www.nutrisurvey.de/ena2011/). In addition, a sample size that would enable detection of a future increase of 0.26 *z*-scores in mean height-for-age *z*-score (HAZ) in children aged 6–35 mo [20], and a 20% relative reduction of anaemia prevalence in children aged 6–59 mo, following 1 y of nutrition supplementation, was calculated [21] using a published formula for detecting differences between surveys [22]. The larger sample size estimates were selected for each target group, and are described below.

To detect a reduction in stunting prevalence from 39.1% to 29.6%, with 80% power and 10% significance level, in children aged 6–35 mo, a sample of 592 children, aged 6–59 mo, was calculated to be required from each stratum. The calculation assumed a normal distribution for HAZ, with a standard deviation of one, a design effect value of 1.5, a desired precision of 10%, and the assumption that 60.6% of children aged 6–59 mo would be 6–35 mo of age.

For women, a target sample size of 574 non-pregnant women, from each stratum, was calculated, based on an expected prevalence of BMI≥25 of 47%, a design effect of 1.5, and a desired precision of 5%.

Households were defined as a group that shared meals and slept under the same roof. An estimated 508 households per camp were needed to reach the required sample size, assuming 1.2 children aged 6–59 mo per household, and a 3% non-response rate. For women, 328 households were estimated required, assuming 1.8 women of childbearing age per household. All eligible children and women present in each household were sampled.

To determine the appropriate number and size of the survey clusters, a pilot field data collection exercise was conducted following training of health and nutrition workers. It was decided to set the cluster size equal to the number of households that a survey team could complete in one day. This resulted in a design using 30 clusters of 17 households in each stratum.

### Survey Teams and Sampling Methods

The survey was carried out by ten survey teams (four members each) and four supervisors. A two-stage sampling method was used [19]. In the first stage, clusters were allocated within the enumeration areas using probability proportional to size sampling. The enumeration area's size was obtained from population estimates available from UNHCR. In the second stage, a random direction was selected from the centre of each enumeration area; all households laying in that direction to the edge of the enumeration area were numbered, and one was selected at random. All remaining households were then sequentially selected by choosing the next-nearest household to the right. To ensure all target members were sampled, households were revisited before leaving the camp if target members were absent during our visit. If households were found empty, they were skipped and not replaced.

### Data and Measurements

Children's date of birth was recorded from health cards. If cards were absent, caregivers were asked to recall the date. Women were asked their age in years, and whether they were currently pregnant and/or lactating. Those reporting either status were excluded from anthropometric measurements.

Weight was measured to the nearest 0.1 kg using a digital scale (Seca 876, Seca). Children were weighed without clothes, and those unable to stand were weighed with the caregiver, using the mother/child function of the scale. Women were asked to remove all accessories, shoes, and excess clothing before being weighed.

Height (or recumbent length for children aged <2 y or measuring <87 cm) was measured to the nearest 0.1 cm using a portable stadiometer (ShorrBoard, Shorr Productions). BMI was obtained by dividing weight (in kilograms) by height (in metres) squared.

WC is considered by some a better predictor of metabolic risk for the individual than BMI [23]. Women's WC was measured using a body measuring tape (model WM02, Chasmors) to the nearest 0.1 cm. The measurement was taken parallel to the floor at the umbilical level, with women wearing no or only light clothing around the waist area.

Presence of bilateral pitting oedema in children was recorded if an imprint remained in both feet after pressing for 3 s.

### Data Computation and Outlier Detection

For children, all anthropometric measurements were computed into *z*-scores—weight-for-age *z*-score (WAZ), HAZ, weight-for-height *z*-score (WHZ), and BMI-for-age *z*-score (BAZ)—using 2006 World Health Organization Growth Standards [24] and ENA for SMART software. Out of range values were defined using a flexible exclusion range criterion (±4 *z*-scores from the observed mean for all indicators, with a maximum HAZ of +3), flagged, and excluded from the analyses [25].

For women, the BACON algorithm, a tool for outlier detection of related variables (i.e., weight, height, and WC), was used to flag and exclude outliers from analyses [26]. Using the 2007 World Health Organization Growth References [27], women's height measurements were computed into HAZ following a procedure similar to one previously described [28]. For women aged ≤19 y, reported age was used for computing HAZ; for women older than 19 y, HAZ was computed assuming an age of 19 y.

Only children aged 6–59 mo and non-pregnant, non-lactating women were included in the analyses and reported in this study.

### Individual Classifications

In children, acute malnutrition (based on WHZ and/or the presence of oedema), stunting (based on HAZ), and underweight (based on WAZ) were defined and classified as global/total (<−2), moderate (<−2 but ≥−3), and severe (<−3) [19]. Overweight was defined as BAZ>2 but ≤3, and obesity as BAZ>3 [29].

For women, stunting was classified as total (<−2), moderate (<−2 but ≥−3), and severe (<−3); underweight was defined as BMI<18.5 kg/m^2^, overweight as BMI≥25 but <30 kg/m^2^ and obesity as BMI≥30 kg/m^2^. Metabolic risk by central obesity was defined as increased (WC≥80 but <88 cm) or substantially increased (WC≥88 cm) [25].

Although both women and children were classified as underweight based on international standards [25], it is important to note that this classification has different meanings for each group, i.e., it represents thinness in the former and a low weight for age in the latter.

### Household Selection and Classification

All sampled households were initially classified as to whether or not they contained cases of (1) under-nutrition in children (GAM: WHZ<−2 and/or oedema; stunting: HAZ<−2; underweight: WAZ<−2) or in women (stunting: HAZ<−2, underweight: BMI < 18.5 kg/m^2^); or (2) overweight in children (BAZ>2) or women (overweight: BMI≥25 kg/m^2^; central obesity: WC≥80 cm).

Next, to quantify the proportion of households with the double burden of malnutrition, households were selected and classified using a modified method previously described [11]. First, households were selected if at least two selected target members were measured. Second, households were classified into one of four groups: (1) undernourished household, i.e., at least one individual presenting with some form of under-nutrition but none presenting with overweight; (2) overweight household, i.e., at least one individual presenting with overweight but none presenting with under-nutrition; (3) double burden household, i.e., at least one individual presenting with some form of under-nutrition and one individual presenting with overweight; (4) normal household, i.e., no individuals presenting with under-nutrition or overweight.

We extended the standard definition of double burden by using WC, considered by some a better predictor of metabolic risk than BMI [23], for classifying the presence of overweight in households. Thus, households were additionally classified as described above but using central obesity instead of overweight in women.

### Statistical Methods

All statistical analyses were carried using Stata (Stata IC release 11, StataCorp). To check for sample bias, initial comparisons were carried out between strata for mean age in both target groups, and for the proportion of males in children.

Means and proportions were calculated using Stata's *svy* function, with each strata sample weighted according to estimated camp population size. We used Pearson's chi-squared to test for association between the presence of overweight and underweight in households.

## Results

### Household Characteristics

Table 1 describes the demographics and number of households sampled in each stratum. A total of 120 clusters with 2,044 households were sampled. The number of households sampled per stratum was similar. Overall, 98.1% of households consented, and 1.2% refused participation, while 0.7% were found to be empty. The absentee rates for children were 3.3%, 10.7%, 5.4%, and 10.0%, and for women were 2.5%, 13.7%, 7.9%, and 15.2%, in Awserd, Dakhla, Laayoune, and Smara camp, respectively.

**Table 1:** Household demographics in the four surveyed Western Sahara refugee camps (strata).

Camp	Households				Children <5 y		Women aged 15–49 y	
	Sampled	Consented	Refused	Empty	Total	6–59 mo	Total	Non-Pregnant, Non-Lactating
Awserd	510	506	2	2	408	366	635	381
Dakhla	510	489	14	7	444	418	719	460
Laayoune	511	505	6	0	392	349	626	416
Smara	513	505	3	5	514	475	768	524
Total	2,044	2,005	25	14	1,758	1,608	2,748	1,781

### Sample Characteristics

For children <5 y, date of birth was missing in four (0.2%); 146 (8.3%) were aged <6 mo, and 91.5% were aged 6–59 mo. For women of childbearing age, age was missing in 15 (0.5%); 26 (0.9%) had no pregnancy or lactating status recorded, 318 (11.6%) were classified as pregnant, 612 (22.1%) as lactating, and 64.8% as non-pregnant, non-lactating.

Summaries of age distributions for both target groups are presented in Table 2, in addition to the proportion of male children sampled, per stratum. No differences between strata were found in mean age and proportion of male children. Likewise for women, no differences in mean age were observed.

**Table 2:** Comparison of sample characteristics between the four surveyed Western Sahara refugee camps (strata).

Characteristic	Camp				Total (*n* = 1,608)
	Awserd (*n* = 366)	Dakhla (*n* = 418)	Laayoune (*n* = 349)	Smara (*n* = 475)	
**Children (6–59 mo)**					
Male sex (percent [95% CI])	50.0 (45.0–55.0)	52.2 (46.4–57.9)	51.9 (47.7–56.1)	51.6 (46.8–56.4)	51.4 (48.9–53.9)
Age (months [95% CI])	28.8 (27.3–30.4)	31.3 (29.7–32.9)	29.9 (28.1–31.6)	31.0 (29.4–32.7)	30.3 (29.4–31.1)
6–17 mo (percent)	31.1	27.5	29.2	25.7	28.2
18–29 mo (percent)	24.0	23.7	24.1	25.3	24.3
30–41 mo (percent)	21.0	17.2	22.1	21.3	20.3
42–53 mo (percent)	15.0	21.5	17.8	17.1	17.9
54–59 mo (percent)	8.7	10.0	6.9	10.7	9.3
**Women** a **(15–49 y)**					
Age (years [95% CI])	30.3 (29.5–31.2)	30.8 (29.6–32.1)	30.5 (29.8–31.4)	30.1 (29.3–31.0)	30.4 (30.0–30.9)
15–24 y (percent)	36.2	36.5	35.6	38.7	36.9
25–34 y (percent)	29.9	27.0	28.8	24.4	27.3
35–44 y (percent)	20.5	19.3	19.7	24.2	21.1
44–49 y (percent)	13.4	17.2	15.9	12.6	14.7

a Non-pregnant, non-lactating women.

95% CI, 95% confidence interval.

### Prevalence of Under-Nutrition, Overweight, Obesity, and Central Obesity

Of the total sample of children selected for analysis (*n* = 1,608), 2.7%, 6.8%, 1.0%, and 4.5% had flagged values for WHZ, HAZ, WAZ, and BAZ, respectively. In addition, 1.5%, 0.7%, 0.5%, and 0.6% had values missing for each of the above indicators, respectively.

Overall, in children, the main form of under-nutrition observed in the camps was stunting, followed by underweight and, lastly, GAM (Table 3). The prevalence of overweight and obesity, as indexed by BAZ, was low.

**Table 3:** Prevalence of nutrition indicators in children and women.

Indicator/Variable	*n*	Mean ± SD	Prevalence (95% CI)
**Children (6–59 mo) (** ***n*** ** = 1,608)**			
**Acute malnutrition**			
Mean WHZ	1,541	−0.38±1.24	
Moderate (WHZ<−2 but ≥−3)			6.3 (5.1–7.5)
Severe (WHZ<−3 and/or oedema)			2.8 (2.0–3.7)
Global (WHZ<−2 and or oedema)			9.1 (7.6–10.7)
**Stunting**			
Mean HAZ	1,488	−1.29±1.24	
Moderate (HAZ<−2 but ≥−3)			20.9 (18.7–23.2)
Severe (HAZ<−3)			8.2 (6.7–9.6)
Total (HAZ<−2)			29.1 (26.4–31.9)
**Underweight**			
Mean WAZ	1,584	−1.00±1.18	
Moderate (WAZ<−2 but ≥−3)			13.5 (11.8–15.2)
Severe (WAZ<−3)			5.1 (3.8–6.4)
Total (WAZ<−2)			18.6 (16.5–20.8)
**Overweight and obesity**			
Mean BAZ	1,527	−0.25±1.23	
Overweight (BAZ>2 but ≤3)			2.4 (1.6–3.3)
Obesity (BAZ>3)			0.8 (0.4–1.3)
**Women** a **(15–49 y) (** ***n*** ** = 1,781)**			
**Stunting**			
Mean HAZ	1,700	−1.07±0.93	
Moderate (HAZ<−2 but ≥−3)			13.0 (11.2–14.7)
Severe (HAZ<−3)			1.9 (1.1–2.7)
Total (HAZ<−2)			14.8 (12.9–16.8)
**Underweight, overweight, and obesity**			
Mean BMI (kilograms/metre^2^)	1,699	26.1±5.2	
Underweight (BMI<18.5)			5.1 (4.0–6.2)
Overweight (BMI≥25 but <30)			31.8 (29.6–34.0)
Obesity (BMI≥30)			21.9 (19.6–24.2)
Overweight or obesity (BMI≥25)			53.7 (51.0–56.4)
**Metabolic risk by central obesity**			
Mean WC (centimetres)	1,689	87.4±12.4	
Increased (WC≥80 but <88)			23.5 (21.3–25.7)
Substantially increased (WC≥88)			47.9 (45.2–50.6)
Central obesity (WC≥80)			71.4 (68.7–74.1)

a Non-pregnant, non-lactating women.

95% CI, 95% confidence interval; SD, standard deviation.

Of the total sample of women selected for analysis (*n* = 1,781), 3.3% of the anthropometric values were considered outliers. In addition, 1.3% and 1.9% of the women had BMI and WC values missing, respectively.

**Figure 1:**
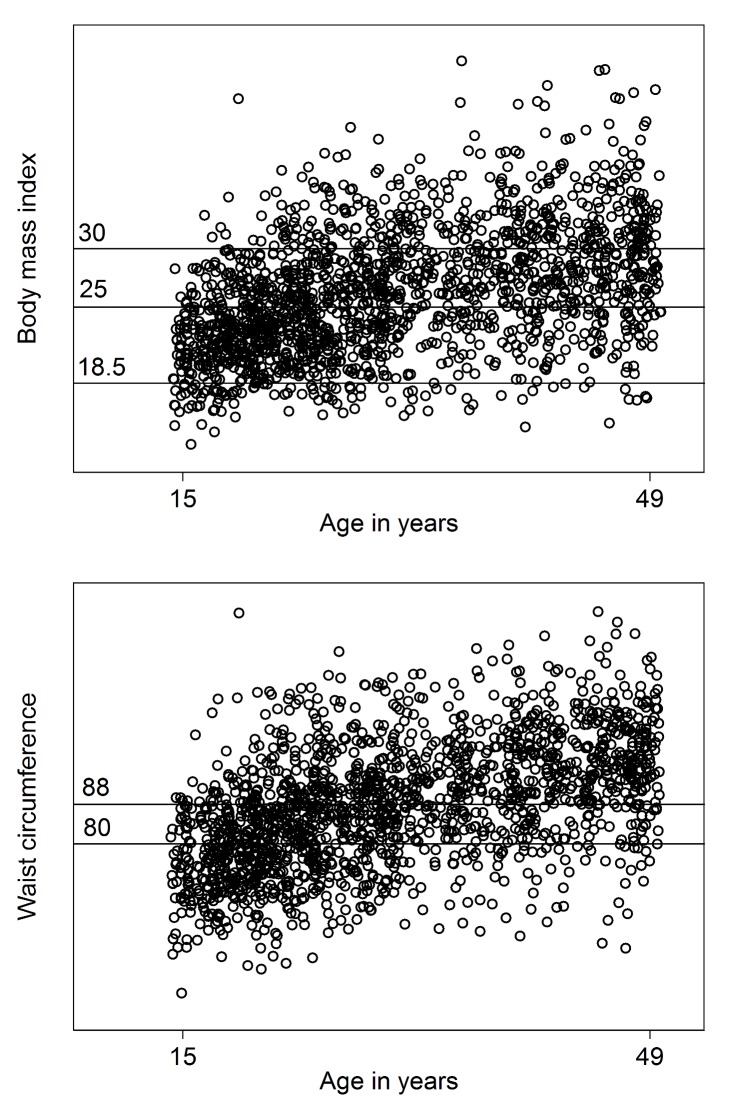
Overweight, obesity, and central obesity among refugee women by age. Scatterplot of the relationship between BMI (in kilograms/metre^2^; linear regression coefficient 0.22, constant 19.3) and WC (in centimetres; linear regression coefficient 0.60, constant 69.0) with age among Western Sahara refugee women.

For women, stunting was the main form of under-nutrition observed (Table 3). Underweight prevalence was low compared with the prevalence of overweight and obesity, which, when combined, affected over half the population. Likewise, the overall prevalence of metabolic risk as defined by central obesity was very high (over 70%). Figure 1 shows that both obesity and central obesity increase with age. A substantially increased metabolic risk due to central obesity, as measured by WC, appears at a markedly younger age than does obesity, as measured by BMI. Both indices show a high prevalence at later ages. The two indices were also highly correlated (*r* = 0.8, *p*<0.01).

### Proportion of Households Presenting Cases of Under-Nutrition, Overweight, or Central Obesity

**Figure 2:**
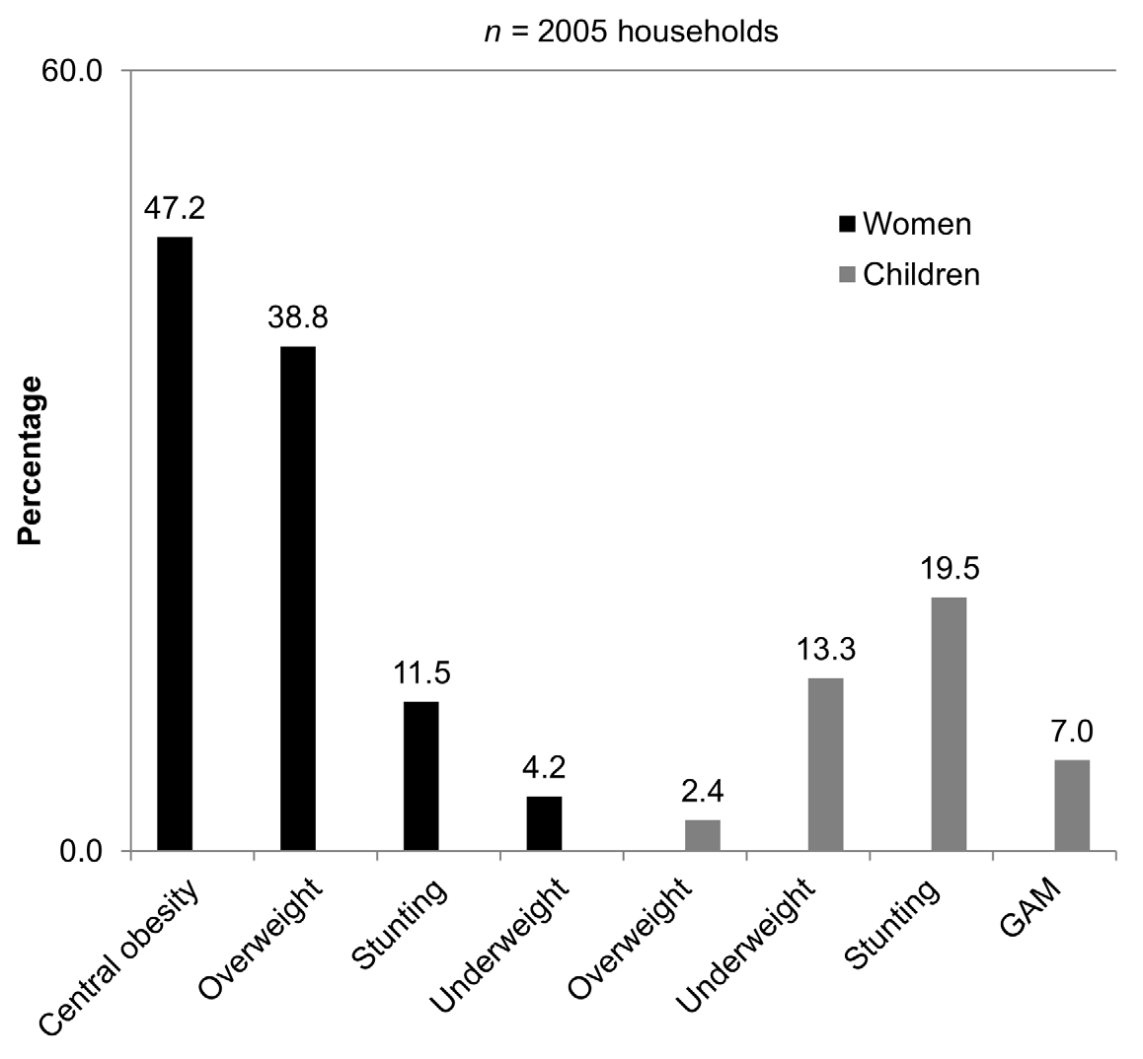
Malnutrition in refugee households. Proportions of households with a member affected by malnutrition in women and children, Western Sahara refugee camps.

Figure 2 shows the proportion of households presenting cases of either some form of under-nutrition or overweight or central obesity, by target group, based on the total number of households sampled (*n* = 2,005). Similar to the pattern observed using individual-level data, the most common type of under-nutrition affecting households was stunting and underweight in children, followed by stunting in women. Overall, 37.8% of households presented at least one case classified with some form of under-nutrition in any target group.

Regarding overweight and central obesity in women, central obesity affected a greater proportion of households than overweight. The proportion of households with children classified as overweight was low. Overall, a total of 53.5% of households had at least one case of either overweight or central obesity, in any target group.

### Proportion of Households Affected by the Double Burden of Malnutrition

**Figure 3:**
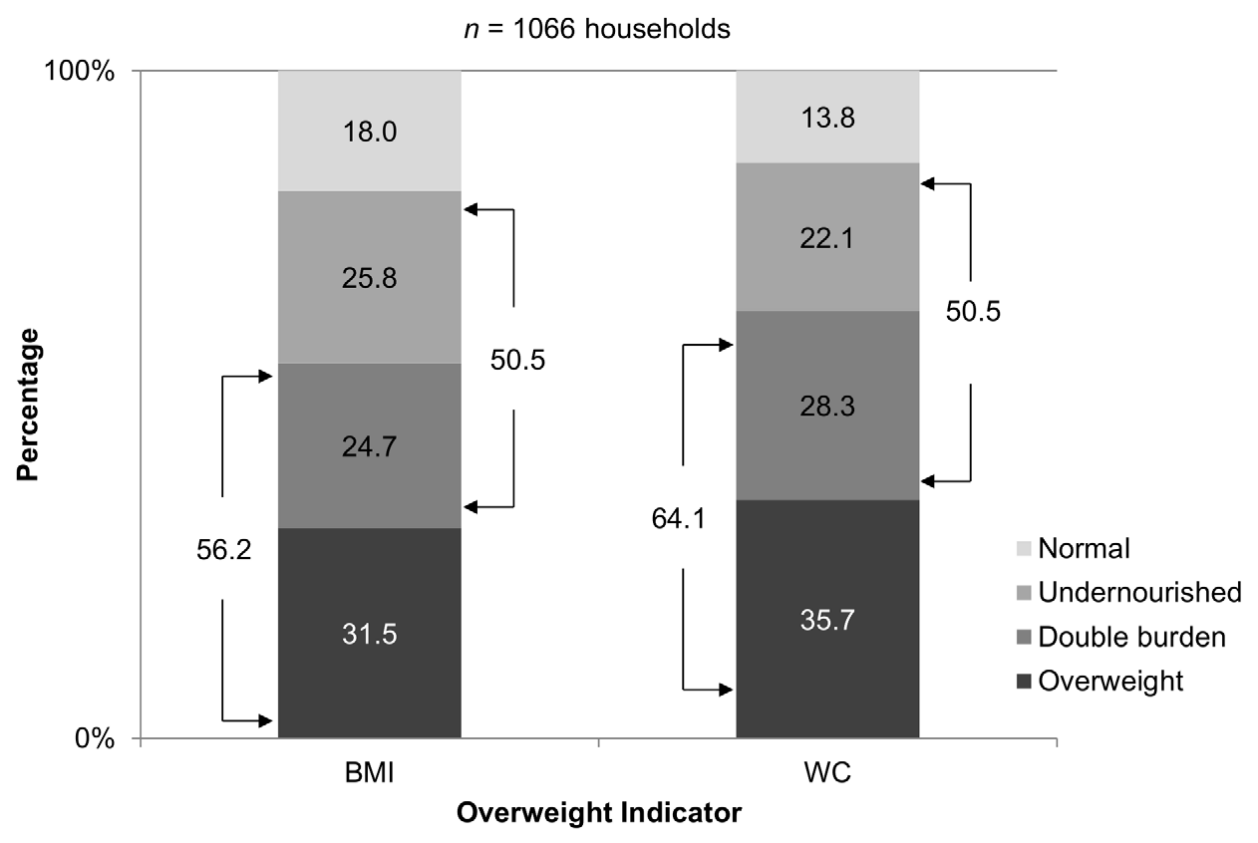
Double burden of malnutrition in refugee households. Proportion of households classified as normal, double burden, overweight, and undernourished in Western Sahara refugee camps. Overweight and the double burden in each stacked bar is based on two different indicators used to classify either obesity (BMI) or central obesity (WC).

Our results showed a negative association between the presence of cases with under-nutrition and the presence of cases with overweight in households (*r* = −0.15, *p*<0.01). The proportion of households classified as undernourished, overweight, double burden, or normal is shown in Figure 3. Based on the households where at least two target group members were measured (*n* = 1,006) and using BMI to classify overweight in women, about one in three households were classified as overweight. The proportion of households classified as undernourished or as double burden was about one in four for each classification, while only 18% of households were classified as normal.

Similar results were observed if overweight classification in women was based on central obesity (WC≥80 cm), although the proportions of households classified as overweight or double burden were slightly greater than those observed using BMI (Figure 3).

## Discussion

To our knowledge, this is the first detailed study of the prevalence and the coexistence of under-nutrition and overweight in a protracted refugee setting where the population has not experienced economic development and is dependent on food assistance for survival. Our results demonstrate that both stunting (in children and women) and obesity (in women) are highly prevalent among Sahrawi refugees, with central obesity being even more prevalent and appearing at a younger age in women than obesity. Second, more households were affected by overweight and central obesity than by under-nutrition, although the latter affected over one-third of households. Third, an important proportion of refugee households, one in four, are affected by the double burden of malnutrition. The results raise crucial and challenging issues for the design of refugee assistance programmes, and the future provision of care for obesity-associated co-morbidities among Sahrawi refugees and other similar populations.

At the proximate level, how could a population that was previously nomadic, possibly experiencing chronic energy insufficiency, have developed the observed high levels of overweight and obesity while living in refugee camps in the absence of economic development? Various factors previously suggested to be associated with obesity among Sahrawi women living in Western Sahara urban centres [30] could possibly also affect those living as refugees.

One factor is that the Sahrawi were traditionally nomadic and culturally associate larger bodies with wealth and beauty, thus fattening practices involving periods of ritual overfeeding, and the use of appetite enhancers and traditional medication (suppositories composed of a mix of dates, seeds, and medicinal plants that are believed to increase peripheral fat accumulation), were common among Sahrawi [31]. Urbanisation has possibly created synergy between these customs and the adoption of processed foods and modern medicines, thereby increasing the likelihood of obesity [31]. Such a synergy might also affect those living as settled refugees, as they depend on food assistance and have limited access to local markets in Algeria.

Another factor is an excessive sugar consumption habit among the Sahrawi [30]. One example is found in the frequent and widespread consumption of green tea (with an average reported consumption among refugees of three servings of 30 ml each, three times a day [32]), which is usually prepared adding about five teaspoons of sugar for each teaspoon of green tea leaves [33]. Sugary drinks are suggested to be among the main drivers of a rapid increase of obesity [34]. Lastly, urbanised Sahrawi women with high BMI values have been found to walk significantly less than those with normal BMI values, thereby reducing their energy expenditure [30].

These factors help to partially explain the high prevalence of overweight in this population; however, they are complemented by other factors affecting refugees living in the camps, which at a more ultimate level help explain the high prevalence of both under-nutrition and overweight in this population. Importantly, some factors that are associated with under-nutrition in early life appear to increase susceptibility to overweight in later life (see the thrifty phenotype hypothesis [35] and the developmental origins of obesity hypothesis [36]). Regarding our study population, both nutritional deficiencies and food insecurity, which, as observed in our findings, often result in wasting and stunting in early life, are also associated with subsequent obesity [37]. The underlying mechanisms are still being established. For instance, studies from shantytowns in Brazil have suggested that stunted children have impaired fat oxidation capacity, a risk factor for obesity [38], although it is not known if this developmental adaptation occurs in other populations. Programming of leptin receptors in the brain is another potential mechanism receiving attention [37]. There is also a growing understanding that individuals experiencing under-nutrition early in life are more susceptible to developing obesity by subsequent exposure to refined, carbohydrate-rich diets and high sugar intake [39], features characteristics of this population's diet.

One crucial aspect is that Sahrawi refugees are dependent on food assistance to cover most of their nutritional needs and thus lack agency over their food system. A typical food assistance basket for this population will often be rich in starchy foods (refined grain cereals, pulses, and blended foods) and sugar. The refugee food assistance package typically contains low quantities, if any, of fresh or dried vegetables and fruit, therefore providing a low-diversity diet [40]. Recent evidence suggests that a low-diversity diet is related to obesity and associated co-morbidities [41], as well as being associated with nutritional deficiencies [42]. In other words, the quality of the diet deriving from the food assistance currently provided may be implicated in both nutritional extremes. Likewise, a lack of control over the broad socioeconomic context, and thus the food system, has recently been suggested to be related to both under-nutrition and obesity [43].

Historically, a high prevalence of obesity, as observed among Sahrawi refugee women, is commonly described among groups that have suffered from severe cultural and economic disruptions with prolonged food insecurity, followed by a rapid transition to more refined foods [44]. A well-known case involves the Pima Amerindians, who were confined on reservations and became dependent on food assistance for generations whilst undergoing a rapid transition from a nomadic to a settled lifestyle. This population now has unusually high levels of obesity and type 2 diabetes [44].

There are various strengths and innovations in this study. Data collection followed a robust and detailed nutrition survey design using internationally recognised protocols for household sampling. This allowed for assessment of nutritional status at both individual and household levels. In addition, to our knowledge, this is the first time obesity and the double burden of malnutrition were assessed using a measure of central obesity, as well as BMI.

However, there are some limitations. First, assessing obesity via BMI and WC presents methodological challenges, as both indicators are affected by variations in lean mass [45],[46], while the former is also affected by body proportions [47], and the latter is not normalised for overall size variance. At present, it is widely acknowledged that BMI and WC are strong predictors of overall mortality and metabolic syndrome morbidity above the cut-off values selected in our analysis [48],[49]; however, our results could potentially represent an underestimation of the real burden of disease, given the frequent suggestion of lowering the cut-off values of both indicators for non-Western populations [50].

In addition to methodological challenges, women wore clothes during weight measurement, which could have increased the overweight prevalence. However, this is unlikely to change the findings, as the prevalence of BMI≥25 kg/m^2^ was only 2.6 percentage points lower after deducting 1 kg for women's clothing during a sensitivity analysis.

Further, we lack additional data that would allow further interpretation of our results. For example, no anthropometric data were obtained for men or other household members, and therefore we could not evaluate their nutritional status. While females are often more at risk of obesity, as demonstrated by the slightly greater secular changes in females' BMI that have occurred worldwide in the last 30 years [3], future work on adult males and other population groups is needed. Likewise, no data were collected that would allow us to narrow our analysis to mother/child biological pairs.

We also did not collect clinical or biochemical markers of metabolic syndrome, and therefore could not ascertain the associated burden of disease in this population. However, data from urbanised Sahrawi women, with comparable levels of central obesity (75%), showed that 16.3% had metabolic syndrome and 28.6% were hypertensive [30]. These proportions are likely to be either similar or greater for Sahrawi refugees because of the added burden of under-nutrition in early life [51].

As no socioeconomic data were collected, we could not assess the role of known social determinants of obesity and under-nutrition, such as poverty or economic and gender inequalities [52]. We feel that gender inequalities, within this population, could partially explain some of the high prevalence of obesity observed among women [52], but economic inequalities and social strata are less likely to play a role in this setting, given the lack of economic development and the widespread reliance on food assistance for survival.

Lastly, as observed in Table 1, more households in the Dakhla camp refused to participate in the survey than in all the other camps. We feel this to be unlikely to bias our results, given the high prevalence of stunting and obesity observed in our results and the low proportion of households that refused (2.7% within that stratum).

Overall, our study highlights an evolving need to focus more effort on NCDs in protracted refugee settings, particularly on obesity, associated co-morbidities, and the double burden of malnutrition described here. The high prevalence of obesity in this Sahrawi refugee population should not be assumed to imply that the population receives excessive or even adequate nutrition. Both under-nutrition and overweight may be considered as alternative forms of malnutrition, where the diet is suboptimal for health [37]. Our data demonstrate that obesity coexists with stunting in this population, and the diet currently provided may underpin both nutritional problems. Additionally, a comprehensive approach to address these issues is needed, rather than the palliative approach currently recommended [53]. This raises numerous challenges.

First, the emergence of obesity and the double burden of malnutrition has serious implications for how international organisations should plan and provide assistance, especially for those exposed to conflict or displacement of protracted duration. For example, food assistance policies need to be revised and adapted, as those currently designed to meet population minimum needs during an acute emergency will need to consider their potential contribution to the later development of NCDs. Additionally, efforts are needed to promote long-term food security and higher nutrition adequacy in protracted emergencies. The actions needed range from improved food security assessments, with special focus on diversity within food groups, to provision of cash or vouchers, to community involvement in sustainable livelihood programmes such as gardening and small-scale business. UNHCR will need to work with the World Food Programme and other organisations on this issue. The Sahrawi refugees have been residing in camps since 1975. Generations of adults from birth have received food assistance as their main source of food. Their children are now the second or third generation exposed to a consistently low-quality diet. The intergenerational impact of this exposure is of serious concern in this and similar protracted emergencies [51].

Second, efforts are needed to evaluate and monitor the health impact of obesity and the double burden in refugee situations. Obesity and NCDs should be routinely included in nutrition and health assessment exercises in protracted refugee settings, and should be incorporated into the UNHCR Health Information System database.

Third, the development of appropriate and effective behaviour change interventions to prevent and tackle obesity in these contexts will need innovative approaches. These will require health personnel and community participation in the identification of needs and implementation of solutions. Additionally, a detailed economic assessment is needed to correctly evaluate the resources needed for prevention and treatment.

Lastly, careful policy and advocacy work will be required to convey the complexity of the situation, and to ensure that continued support for life-saving food assistance programmes and the tackling of under-nutrition and nutritional deficiencies is not jeopardised as the threat of obesity to refugee health receives the attention it deserves.

## Supporting Information

Alternative Language Abstract S1 French translation of the abstract by David Beran and Aurore Virayie.
(DOC)

Alternative Language Abstract S2 Spanish translation of the abstract by Carlos S. Grijalva-Eternod and Alejandra J. Cantoral-Preciado.
(DOC)

Alternative Language Abstract S3 Arabic translation of the abstract by Elham Aljaaly and AlBandary AlJameel.
(DOC)

## Abbreviations

BAZ: body-mass-index-for-age *z*-score
BMI: body mass index
GAM: global acute malnutrition
HAZ: height-for-age *z*-score
NCD: non-communicable disease
UNHCR: United Nations High Commissioner for Refugees
WAZ: weight-for-age *z*-score
WC: waist circumference
WHZ: weight-for-height *z*-score
