# Supplementary material for: The Double Burden of Obesity and Malnutrition in a Protracted Emergency Setting: A Cross-Sectional Study of Western Sahara Refugees
Source: PLoS Med. 2012 Oct 2;9(10):e1001320. doi: 10.1371/journal.pmed.1001320 (PMC3462761; doi:10.1371/journal.pmed.1001320)
Supplement: Alternative Language Abstract S1 — French translation of the abstract by David Beran and Aurore Virayie. (DOC) [file pmed.1001320.s001.doc]

Translation of the abstract (The Double Burden of Obesity and Malnutrition in a Protracted Emergency Setting: A Cross-Sectional Study of Western Sahara Refugees) into French by David Beran^1^ & Aurore Virayie^2^

^1^ Division of International and Humanitarian Medicine, Faculty of Medicine, University of Geneva ([david.beran@unige.ch](mailto:david.beran@unige.ch)). ^2^ Independent consultant, Martinique ([aurore.virayie@gmail.com](mailto:aurore.virayie@gmail.com)).

**Le double fardeau de l’obésité et de la malnutrition au cours d’une situation d'urgence prolongée: étude transversale au sein des réfugiés du Sahara Occidental**

Carlos S Grijalva-Eternod^1,2^, Jonathan CK Wells^3^, Mario Cortina-Borja^4^, Nuria Salse-Ubach^5^, Mélody C Tondeur^2^, Carmen Dolan^3^, Chafik Meziani^6^, Caroline Wilkinson^7^, Paul Spiegel^7^, Andrew J Seal^1,2^

^1^ Centre for International Health & Development, UCL Institute of Child Health, London, UK. ^2^ Emergency Nutrition Network, Oxford, UK. ^3^ MRC Childhood Nutrition Research Centre, UCL Institute of Child Health, London, UK. ^4^ MRC Centre of Epidemiology for Child Health, UCL Institute of Child Health, London, UK. ^5^ Independent Consultant, Barcelona, Spain. ^6^ Tindouf Sub-Office, United Nations High Commissioner for Refugees, Tindouf, Algeria. ^7^ Public Health and HIV Section, Division of Programme Support and Management, United Nations High Commissioner for Refugees, Geneva, Switzerland

**Contexte**

On sait que les ménages de groupes vulnérables vivant en situation de transitions épidémiologiques sont affectés de façon concomitante par la sous-nutrition et l'obésité. On ignore encore cependant dans quelles proportions ce double fardeau affecte les populations réfugiées, dépendantes de l'aide alimentaire. Nous avons évalué le double fardeau de la malnutrition chez les réfugiés du Sahara Occidental, qui vivent en situation d'urgence prolongée.

**Méthodes et résultats**

Nous avons effectué une enquête nutritionnelle stratifiée dans quatre camps de réfugiés du Sahara Occidental en Algérie en octobre et novembre 2010. Nous avons échantillonné 2005 ménages et collecté des mesures anthropométriques (poids, taille, et tour de taille) chez 1608 enfants (6-59 mois) et 1781 femmes (15-49 ans). Nous avons estimé les prévalences de malnutrition aiguë globale (MAG), retard de croissance, insuffisance pondérale et de surpoids chez les enfants. Chez les femmes, ont été évalués: le retard de croissance, l’insuffisance pondérale, le surpoids et l'obésité centrale. Pour mesurer l’importance de la malnutrition au sein des ménages, ceux-ci ont d’abord été identifiés selon le type de malnutrition qu’ils présentaient. Ces ménages ont ensuite été classés comme suit : sous-alimentés, en surpoids, ou touchés par le double fardeau s’ils présentaient des membres souffrant de sous-nutrition ou de surpoids, de façon isolée ou combinée. La prévalence de la MAG chez les enfants était de 9,1% ;  29,1% d’enfants souffraient d’un retard de croissance, 18,6% présentaient une insuffisance pondérale, et 2,4% étaient en surpoids, tandis que chez les femmes 14,8% présentaient un retard de croissance, 53,7% étaient en surpoids ou obèses, et 71,4% souffraient d’obésité centrale. L'obésité centrale (47,2%) et le surpoids (38,8%) chez les femmes ont touché une proportion plus élevée de ménages que la MAG (7,0%), le retard de croissance (19,5%), ou encore l’insuffisance pondérale (13,3%) chez les enfants. Globalement, les ménages classés comme étant en surpoids (31,5%) étaient les plus nombreux, suivis par les ménages considérés comme sous-alimentés (25,8%), puis ceux touchés par le double fardeau (24,7%).

**Conclusions**

Le double fardeau de la malnutrition est très répandu dans les ménages réfugiés du Sahara Occidental. Les résultats mettent en évidence la nécessité de se focaliser davantage sur les maladies non transmissibles au sein de cette population et d’intégrer de façon équilibrée prévention et gestion de l’obésité avec les interventions de lutte contre la sous-nutrition.
