# Supplementary material for: The Double Burden of Obesity and Malnutrition in a Protracted Emergency Setting: A Cross-Sectional Study of Western Sahara Refugees
Source: PLoS Med. 2012 Oct 2;9(10):e1001320. doi: 10.1371/journal.pmed.1001320 (PMC3462761; doi:10.1371/journal.pmed.1001320)
Supplement: Alternative Language Abstract S2 — Spanish translation of the abstract by Carlos S. Grijalva-Eternod and Alejandra J. Cantoral-Preciado. (DOC) [file pmed.1001320.s002.doc]

Translation of the abstract (The Double Burden of Obesity and Malnutrition in a Protracted Emergency Setting: A Cross-Sectional Study of Western Sahara Refugees) into Spanish by Carlos S Grijalva-Eternod^1^ & Alejandra J Cantoral-Preciado^2^

^1^ Centre for International Health & Development, UCL Institute of Child Health, London, UK ([c.eternod@ucl.ac.uk](mailto:c.eternod@ucl.ac.uk)). ^2^ Dirección de Estadística del Centro de Investigación en Evaluación y Encuestas, Instituto Nacional de Salud Pública, Cuernavaca, México ([Alejandra.cantoral@insp.mx](mailto:Alejandra.cantoral@insp.mx))

**La Doble Carga de Obesidad y Malanutrición en una Situación de Emergencia Prolongada: Un Estudio de Corte Transversal en Refugiados del Sáhara Occidental**

Carlos S Grijalva-Eternod^1,2^, Jonathan CK Wells^3^, Mario Cortina-Borja^4^, Nuria Salse-Ubach^5^, Mélody C Tondeur^2^, Carmen Dolan^3^, Chafik Meziani^6^, Caroline Wilkinson^7^, Paul Spiegel^7^, Andrew J Seal^1,2^

^1^ Centre for International Health & Development, UCL Institute of Child Health, London, UK. ^2^ Emergency Nutrition Network, Oxford, UK. ^3^ MRC Childhood Nutrition Research Centre, UCL Institute of Child Health, London, UK. ^4^ MRC Centre of Epidemiology for Child Health, UCL Institute of Child Health, London, UK. ^5^ Independent Consultant, Barcelona, Spain. ^6^ Tindouf Sub-Office, United Nations High Commissioner for Refugees, Tindouf, Algeria. ^7^ Public Health and HIV Section, Division of Programme Support and Management, United Nations High Commissioner for Refugees, Geneva, Switzerland

**Antecedentes**

Es sabido que los hogares pertenecientes a grupos vulnerables que sufren una transición epidemiológica están afectados de forma concomitante por desnutrición y obesidad. Sin embargo, se desconoce hasta qué punto esta doble carga afecta a poblaciones en situación de emergencia que dependen de asistencia alimentaria. Este estudio evaluó la doble carga de malanutrición entre los refugiados del Sáhara Occidental, que viven en una situación de emergencia prolongada.

**Métodos y Resultados**

Se implementó una encuesta de nutrición estratificada en Octubre-Noviembre 2010 en los cuatro campamentos de refugiados del Sahara Occidental en Argelia. Se encuestó una muestra de 2005 hogares, recogiendo mediciones antropométricas (peso, talla y circunferencia de cintura) en 1608 niños (6-59 meses) y 1781 mujeres (15-49 años). Se estimó la prevalencia de malanutrición aguda global (MAG), baja talla, bajo peso y sobrepeso en niños; y la prevalencia de baja talla, bajo peso, sobrepeso, y obesidad central en mujeres. Para evaluar la carga de malanutrición en los hogares éstos fueron primero clasificados de acuerdo a la presencia de cada tipo de malanutrición. Los hogares fueron subsecuentemente clasificados como afectados por sobrepeso, desnutrición, o por la doble carga, si en éstos se presentaban miembros con desnutrición o sobrepeso ya sea de forma aislada, o en combinación.

La prevalencia de la MAG en los niños fue del 9.1%, el 29.1% sufría de baja talla, el 18.6% tenía bajo peso, y el 2.4% tenía sobrepeso, mientras que para las mujeres el 14.8% sufría de baja talla, el 53.7% tenía sobrepeso u obesidad, y el 71.4% presentaba obesidad central. La obesidad central (47.2%) y sobrepeso (38.8%) en las mujeres afectó a una mayor proporción de hogares, comparado con la MAG (7.0%), baja talla (19.5%), o bajo peso (13.3%) en los niños. En general, los hogares clasificados con sobrepeso (31.5%) fueron los más comunes, seguidos por aquellos clasificados con desnutrición (25.8%), y finalmente con la doble carga (24.7%).

**Conclusiones**

La doble carga de obesidad y malanutrición es altamente prevalente en los hogares de los refugiados del Sáhara Occidental. Nuestros hallazgos destacan la necesidad de prestar una mayor atención en esta población a las enfermedades no transmisibles, y de integrar de forma equilibrada prevención y gestión de la obesidad con intervenciones para la lucha contra la desnutrición.
