# Supplementary material for: The Double Burden of Obesity and Malnutrition in a Protracted Emergency Setting: A Cross-Sectional Study of Western Sahara Refugees
Source: PLoS Med. 2012 Oct 2;9(10):e1001320. doi: 10.1371/journal.pmed.1001320 (PMC3462761; doi:10.1371/journal.pmed.1001320)
Supplement: Alternative Language Abstract S3 — Arabic translation of the abstract by Elham Aljaaly and AlBandary AlJameel. (DOC) [file pmed.1001320.s003.doc]

Translation of the abstract (The Double Burden of Obesity and Malnutrition in a Protracted Emergency Setting: A Cross-Sectional Study of Western Sahara Refugees) into Arabic by Elham Aljaaly^1^ & AlBandary AlJameel^2^

^1^ Centre for International Health & Development, UCL Institute of Child Health, London, UK ([e.aljaaly@ucl.ac.uk](mailto:e.aljaaly@ucl.ac.uk) or [aljaalydiet@yahoo.com](mailto:aljaalydiet@yahoo.com)). ^2^ UCL Epidemiology and Public Health, London, UK ([a.al-jameel@ucl.ac.uk](mailto:a.al-jameel@ucl.ac.uk))

العبء المزدوج للسمنة وسوء التغذية في الحالات الطارئة لفترات طويلة : دراسة مقطعيه على أسر اللاجئين في الصحراء الغربية

Carlos S Grijalva-Eternod^1,2^, Jonathan CK Wells^3^, Mario Cortina-Borja^4^, Nuria Salse-Ubach^5^, Mélody C Tondeur^2^, Carmen Dolan^3^, Chafik Meziani^6^, Caroline Wilkinson^7^, Paul Spiegel^7^, Andrew J Seal^1,2^

^1^ Centre for International Health & Development, UCL Institute of Child Health, London, UK. ^2^ Emergency Nutrition Network, Oxford, UK. ^3^ MRC Childhood Nutrition Research Centre, UCL Institute of Child Health, London, UK. ^4^ MRC Centre of Epidemiology for Child Health, UCL Institute of Child Health, London, UK. ^5^ Independent Consultant, Barcelona, Spain. ^6^ Tindouf Sub-Office, United Nations High Commissioner for Refugees, Tindouf, Algeria. ^7^ Public Health and HIV Section, Division of Programme Support and Management, United Nations High Commissioner for Refugees, Geneva, Switzerland

مقدمة

من المتعارف عليه أن الأسر من الفئات الفقيرة والتي تعاني من التحولات الوبائية تتأثر و بصورة متزامنة من جراء تأثير العبء المزدوج الناتج عن المشاكل الغذائية المرتبطة بالسمنة ونقص التغذية، و حتى وقتنا الحالي لم يتم معرفة مدى هذا التأثير على اللاجئين الذين يعتمدون على المساعدات الغذائية. من أجل ذلك هدفت هذه الدراسة إلى تحليل وتقييم العبء المزدوج لسوء التغذية بين اللاجئين الذين يعيشون في ظروف طارئة سيئة ولفترة زمنية طويلة في مخيمات اللاجئين بمناطق الصحراء الغربية بدولة الجزائر في شمال أفريقيا.

طرق البحث والنتائج

لقد تم عمل مسح طبقي شامل لأربعة من مخيمات اللاجئين بالصحراء الغربية بالجزائر وذلك خلال شهريّ أكتوبر/ نوفمبر من عام ٢٠١٠. وتم أخذ عينة مكونة من ٢٠٠٥ أسرة و أجريت القياسات البشرية (الطول، الوزن، محيط الوسط) ل ١٦٠٨ طفل تراوحت أعمارهم ما بين ٦ إلى٥٩ شهر و ١٧٨١ امرأة تراوحت أعمارهنّ ما بين١٥إلى ٤٩ سنة وتم بموجبه تقدير معدل انتشار سوء التغذية الحاد مقارنة بالنسبة المتعارف عليها بين دول العالم المختلفة (GAM)، قصر القامة ونقص الوزن وزيادته بين الأطفال. وبين السيدات تم أيضا تقدير السمنة المتمركزة في منطقة الوسط بالإضافة إلى القياسات السابقة.

لتقييم العبء المزدوج لسوء التغذية بين الأسر تم التصنيف كالتالي: ١) تصنيف الأسر من حيث وجود كل نوع من سوء التغذية (سمنة، نقص تغذية، قصر قامة،....الخ). ٢) تصنيف الأسر من حيث نسبة زيادة الوزن أونقصه أو وجود الحالتين معا.

ولقد تم التوصل إلى النتائج التالية: ١) أن معدل انتشار سوء التغذية الحاد مقارنة بالنسبة الدولية بين الأطفال كان بنسبة ٩.١ ٪، قصر القامة بنسبة ٢٩.١٪، نقص الوزن بنسبة ١٨.٦٪، وزيادة الوزن بنسبة ٢.٤٪. أما بالنسبة للسيدات فقد بلغت نسبة قصر القامة ١٤.٨٪، وبلغت نسبة زيادة الوزن والسمنة ٥٣.٧٪ وكان من بينهنّ ٧١.٤٪ يعانِنّ من تمركز السمنة في منطقة الوسط.

٢) ولقد وجدنا أن السمنة المتمركزة في منطقة الوسط (٤٧.٢ ٪) و زيادة الوزن (٣٨.٨ ٪) بين السيدات ذات تأثير أعلى على بقية أفراد الأسرة مقارنة بمدى تأثير الأطفال الذين يعانون من انتشار سوء التغذية الحاد (٧.٠ ٪)، قصر القامة (١٩.٥ ٪)، أو الذّين يعانون من نقص الوزن (١٣.٣ ٪) . وبصفة عامة نجد أن الأسر المصنفة بزيادة الوزن والسمنة هنّ الأكثر انتشاراً من غيرهن من الأسر(٣١.٥٪)، تليها الأسر المصنفة بسوء التغذية (٢٥.٨ ٪) و أخيراً الأسر المصنفة بالعبء المزدوج واللاتي تبلغ نسبتهن ٢٤.٧٪.

الخلاصة

إن العبء المزدوج للسمنة ونقص التغذية منتشر بنسبة مرتفعة بين أفراد أسر اللاجئين في الصحراء الغربية. و إن النتائج التي تم التوصل إليها تسلط الضوء على ضرورة التركيزعلى الأمراض غير المعدية لهذه الفئة وتزيد من مدى الحاجة إلى ابتكار أساليب للوقاية من البدانة ووضع سياسات ونظم منفصلة عن تلك الخاصة بمعالجة نقص التغذية حيث أنه رغم وجود حاجة الى معالجة حالات نقص التغذية لهؤلاء اللاجئين نجد أيضاً أن هنالك نسبة كبيرة من بينهم يعانون من السمنة وزيادة الوزن.
